# Effect of bariatric surgery on matrix metalloproteinase activity in patients with morbid obesity

**DOI:** 10.3389/fmed.2026.1836747

**Published:** 2026-06-15

**Authors:** Barbara Choromańska, Piotr Myśliwiec, Jacek Dadan, Alan Tkaczuk, Konrad Wiśniewski, Almantas Maleckas, Anna Zalewska, Małgorzata Żendzian-Piotrowska, Mateusz Maciejczyk

**Affiliations:** 1 First Clinical Department of General and Endocrine Surgery, Medical University of Bialystok, Bialystok, Poland; 2 Department of Surgery, Lithuanian University of Health Sciences, Kaunas, Lithuania; 3 Department of Gastrosurgical Research and Education, Sahlgrenska Academy, University of Gothenburg, Gothenburg, Sweden; 4 Experimental Dentistry Laboratory, Medical University of Bialystok, Bialystok, Poland; 5 Department of Hygiene, Epidemiology and Ergonomics, Medical University of Bialystok, Bialystok, Poland

**Keywords:** matrix metalloproteinases, metabolic syndrome, MMP, obesity, oxidative stress

## Abstract

Pathological remodeling of extracellular matrix was observed in the course of obesity; however, the detailed role of matrix metalloproteinases (MMPs) in the development of obesity and its metabolic complications remain unclear. The aim of the study was to evaluate plasma activity of selected MMPs in individuals with morbid obesity, as well as the impact of laparoscopic sleeve gastrectomy on MMPs’ activity. MMPs’ metabolism between obese individuals without and with metabolic syndrome was compared. The activities of MMPs were measured fluorometrically. Plasma activities of collagenases (MMP-1, MMP-8, and MMP-13), gelatinases (MMP-9), stromelysins (MMP-3 and MMP-10), metalloelastase (MMP-12) and membrane types MMP (MMP-14) was increased in morbidly obese women without metabolic syndrome before bariatric surgery, whereas activities of MMP-1, MMP-3, MMP-10, and MMP-12 were higher in morbidly obese women with metabolic syndrome than in the lean controls. After bariatric treatment, only the activity of some plasma MMPs partially normalized in both obese groups studied. Obesity is associated with increased activities of plasma MMPs. Although bariatric treatment improves MMPs homeostasis of individuals with obesity, it remained disturbed even 12 months after surgery. In general, the MMPs activity MMPs did not differ between obese individuals without and with metabolic syndrome. However, in individuals with metabolic syndrome, disturbances in the activity of MMPs persisted longer after bariatric surgery. Further long-term studies are needed to validate our findings.

## Introduction

Obesity is a multifactorial disease of epidemic proportions (1). Related diseases of obesity such as type 2 diabetes (T2DM), hypertension, cardiovascular diseases, and cancer are major causes of mortality. However, despite multiple studies, the exact mechanism of obesity is still obscure. It is not clear why some obese patients develop metabolic complications, while others do not. One of suggested links between obesity and related diseases are interactions of proteins and hormones (adiponectin leptin, ghrelin, visfatin and resistin) (2, 3). Lipid distribution resulting from increased expression of fatty acid and cholesterol transporter proteins has also been described as a cause of metabolic syndrome (MS) (4). Oxidative and nitrosative stress as well as antioxidant barrier disorders are also been involved in the pathogenesis of obesity and its complications (5). With the progression of metabolic disorders related to obesity, antioxidant mechanisms are reduced, whereas myeloperoxidase activity, NO formation and oxidative and nitrosative protein damage are increased (5, 6). It is emphasized that adipose tissue is not only a place of fat storage, but also a metabolically active organ (7). Both dysfunction and abnormal distribution of adipose tissue can lead to development of obesity and systemic metabolic disorders (8, 9). The following processes play a role in modifying of adipose tissue structure and/or function: adipogenesis, angiogenesis and proteolysis of the extracellular matrix (ECM) (10). Matrix metalloproteinases (MMPs) are main components of proteolytic system. MMPs, by degrading ECM and/or releasing, activating or damaging cytokines and growth factors, contribute to adipose tissue remodeling and thus are implicated in obesity development (11, 12). MMPs induce degradation of ECM collagen components such as fibronectin and laminin, as well as type IV collagen, gelatin and fibrinogen (13). In obesity, abnormal collagen deposition during ECM remodeling is associated with inflammation in adipose tissue and the development of insulin resistance (14, 15). It has been shown that in transgenic mice increased expression of matrix MMP 14 (MMP-14) in obese adipose tissue leads to weight gain, impaired lipid metabolism and insulin resistance (16). In humans, MMP-14 gene polymorphism was associated with obesity and diabetes (17). It has also been described that 1, 2, 3, 7, and 9 matrix MMPs participate in the formation and destabilization of atherosclerotic plaque (13, 18, 19). Additionally, MMP-7 is implicated in the pathophysiology of cardiovascular diseases. Pathological remodeling of the ECM was observed in the course of myocardial infarction and heart failure (20). However, the detailed mechanisms of MMPs involvement in the development of obesity and its metabolic complications remain unclear. Nevertheless, some study suggested that MMP-2 and MMP-9, are crucial factors linking ECM remodeling, inflammation, and metabolic dysfunction. MMP-2 cleaves the extracellular domain of the leptin receptor, disrupting the control of hunger and satiety in the hypothalamus, thus promoting weight gain (21). Increased MMP-9 activity in obesity has been shown to correlate with systemic inflammation (CRP, IL-6, TNF-α) (22, 23). MMP-1 may be involved in adipose tissue remodeling during adipose tissue gain in obesity (24). Data about the influence of bariatric treatment on MMPs is also lacking (25, 26). Bariatric surgery is the most effective method of reducing adipose tissue and metabolic disorders related to obesity (27, 28). Interestingly, Gentile et al. (29) showed reductions in MMP-2 and MMP-9 levels after bariatric surgery, which are markers of ECM remodeling. Can reduction of the pathological processes of adipose tissue modification and the improvement of MMPs homeostasis contribute to the beneficial effect of bariatric treatment? In response to this question and taking into account current knowledge, the aim of our study was to evaluate plasma activity, not only of MMP-1, MMP-2, and MMP-9 but also other less studied matrix metalloproteinases in this topic, in individuals with morbid obesity, as well as the impact of bariatric surgery on MMPs’ activity 1, 3, 6 and 12 months after laparoscopic sleeve gastrectomy. We also compared MMPs’ metabolism between obese individuals without metabolic syndrome and those with metabolic syndrome.

## Materials and methods

The study was performed in 63 women with class 3 obesity (BMI > 40 kg/m^2^), who underwent elective laparoscopic sleeve gastrectomy at the 1st Department of General and Endocrine Surgery at the University Hospital in Bialystok, Poland. The individuals with obesity were classified into two groups: 33 morbidly obese women without metabolic syndrome aged from 33 to 52 (OB) and 30 morbidly obese women with metabolic syndrome aged from 35 to 56 (OB + MS). In OB + MS group 26 patients suffered from hypertension and 13 patients had type 2 diabetes mellitus (T2DM). Patients with hypertension were diagnosed in accordance with World Health Organization (30) whereas T2DM metabolic syndrome (MS) according to the International Diabetes Federation guidelines (31). Systolic and diastolic blood pressure was measured in the non-dominant upper arm two times at few minutes intervals using Diagnosis UA-651 A&D Medical apparatus. The blood samples for investigation were collected prior to (OB 0; OB + MS 0), as well as 1 month (OB 1; OB + MS 1), 3 months (OB 3; OB + MS 3), 6 months (OB 6; OB + MS 6) and 12 months (OB 12; OB + MS 12) after bariatric surgery.

The controls group comprised of 24 healthy lean women (BMI < 25 kg/m^2^) aged from 35 to 55 who had control visits at the Specialist Dental Clinic at the Medical University of Bialystok, Poland. Willing volunteers with reference range of biochemical blood parameters (AST, ALT, creatinine, K^+^, Na^+^, and INR) and blood counts were eligible to this group.

The exclusion criteria for both the control and the study group included malignancy history, acute inflammatory infections, infectious diseases (hepatitis A, B, and C, HIV/AIDS), gout, type 1 diabetes, mucopolysaccharidosis, osteoporosis, autoimmune diseases (Crohn’s disease, ulcerative colitis, Hashimoto’s disease), cardiovascular disorders (other than hypertension in morbidly obese patients with metabolic syndrome), genitourinary and respiratory systems diseases (other than obstructive sleep apnea in obese patients). Chronic smoking, alcohol abuse, as well as pregnancy were also excluded. In the 3-month period preceding the blood samples collection for this study, healthy controls and obese patients declared not taking any vitamins, non-steroidal anti-inflammatory drugs, antibiotics, glucocorticosteroids and antioxidant supplements. Table 1 presents the clinical and laboratory data of the control and study groups.

**TABLE 1:** Clinical and laboratory parameters of the controls (C), morbidly obese women without metabolic syndrome (OB) and morbidly obese women with metabolic syndrome (OB + MS).

	C	OB 0	OB 1	OB 3	OB 6	OB 12	OB + MS 0	OB + MS 1	OB + MS 3	OB + MS 6	OB + MS 12
BMI (kg/m^2^)	23.1 (22.6–23.5)	44.9**** (41.6–48)	40.4**** (36.9–43.1)	36.6**** (33–39.3)	32****## (29.7–34.8)	29.2*#### (27–30.8)	47.2****∼ (45.2–50.6)	41.7****### (40.5–45.9)	39.3****#### (35.8–41.8)	34.4****####∼ (32.5–37.7)	31.6****####∼∼ (29.6–34.9)
WHR	0.71 (0.7–0.72)	0.95**** (0.92–0.99)	0.96**** (0.91–0.99)	0.94**** (0.88–0.98)	0.93**** (0.89–0.97)	0.91**** (0.87–0.95)	0.98****∼ (0.96–0.99)	0.99****∼∼ (0.97–1.01)	0.98****∼∼ (0.95–0.99)	0.97****∼∼ (0.94–0.99)	0.94****##∼ (0.91–0.96)
SBP	120 (110–120)	120 (120–130)	120 (120–125)	120 (120–129)	120 (120–129)	120 (120–120)	130****∼∼ (125–140)	140****∼∼∼∼ (130–140)	135****∼∼∼∼ (130–140)	135****∼∼∼∼ (123–140)	130****∼∼∼∼ (126–139)
DBP	80 (80–80)	80 (80–90)	80 (80–85)	80 (80–85)	80 (80–85)	80 (80–80)	85*** (80–90)	90****∼∼∼∼ (85–90)	90***∼∼∼ (80–90)	85** (80–90)	85***∼∼∼ (80–90)
Glucose (mg/dL)	77 (75–83)	98**** (91–105)	94**** (87–98)	86**## (81–92)	84**## (81–95)	84### (80–91)	106****∼∼ (101–120)	99.5****∼∼∼ (97–112)	97.5**∼∼∼∼ (93–105)	96**∼∼∼ (91–100)	92∼∼∼ (87–97)
HOMA-IR	1.44 (1.34–1.57)	4.26**** (3.68–5.03)	2.98*** (1.73–4.02)	1.98#### (1.38–2.75)	1.88#### (1.3–2.07)	1.67#### (1.01–1.86)	5.62****∼ (4.18–6.59)	5.33****∼∼∼∼ (4.03–6.28)	2.54####∼ (1.9–3.34)	2.04####∼∼ (1.86–2.89)	1.91####∼ (1.73–2.07)
ALT (IU/L)	24 (21–28)	25 (21–33)	24 (18–35)	22 (17–26)	19# (15–25)	18*## (15–23)	27 (19–36)	29 (20–40)	25 (18–31)	21 (20–27)	19# (18–23)
AST (IU/L)	22 (19–26)	19 (17–26)	25 (19–33)	19 (16–26)	18 (16–23)	17.5 (15–26)	23 (18–31)	27 (20–35)	21 (17–28)	20 (16–29)	19 (16–25)
Cholesterol (mg/dL)	175 (166–180)	197** (175–209)	191** (180–205)	177 (155–191)	176 (166–188)	175## (156–183)	209****∼∼ (196–229)	194***# (183–215)	182#### (176–198)	188**###∼ (176–205)	174#### (168–187)
LDL (mg/dL)	118 (115–120)	125 (117–141)	112## (98–120)	109## (100–128)	109### (98–118)	112### (96–118)	144****∼∼∼∼ (132–162)	123####∼ (110–134)	116#### (110–125)	110#### (95–128)	102#### (93–118)
HDL (mg/dL)	60 (56–64)	49**** (41–55)	45**** (38–55)	47**** (42–52)	50*** (44–55)	52 (48–58)	46**** (38–53)	45**** (39–50)	49**** (45–53)	53### (49–60)	56#### (52–62)
TG (mg/dL)	134 (129–138)	130 (110–147)	109* (91–130)	104***# (86–116)	98***### (80–113)	95****### (80–110)	150*∼ (127–190)	140∼∼ (120–175)	137∼∼∼∼ (118–164)	130#∼∼∼∼ (116–142)	107#### (93–125)
CRP (mg/L)	5.5 (5.3–5.7)	8.8 (5.5–13.9)	6.5 (4.8–9.2)	6.1 (3.3–8.2)	4.43### (2.8–6.7)	4.9#### (2.6–5.2)	11.7**** (8.6–14)	8.7****##∼ (6.8–10.2)	7.3**####∼ (6.3–9.3)	6.3####∼ (5.3–7.3)	5.8####∼∼∼ (5.3–6.8)
Fibrinogen (mg/dL)	344 (304–367)	412**** (400–464)	410**** (387–453)	402**** (374–454)	373### (348–410)	346#### (312–387)	443***∼∼ (427–492)	447***∼∼ (411–487)	417***∼ (398–485)	405***∼∼∼ (393–437)	392∼∼∼∼ (374–417)
WBC (10^3^/μL)	7.5 (6.6–8.3)	8.5 (7.6–9.7)	6.6### (6.1–7.5)	5.8*#### (5.5–6.8)	5.9****#### (5.7–7.3)	6.1**#### (4.8–6.9)	9.6**** (8.2–10.6)	7.2##∼ (6.6–8.5)	6.9##∼∼∼ (6.3–9.2)	7.2###∼ (5.9–8.9)	6.9#### (5.2–7.9)
RBC (10^6^/μL)	4.6 (4.4–4.9)	4.7 (4.4–5.1)	4.8 (4.6–5.1)	4.7 (4.5–4.9)	4.7 (4.5–5.1)	4.7 (4.5–4.9)	4.6 (4.2–5.1)	4.8 (4.6–5.2)	4.8∼ (4.6–5.1)	4.8 (4.5–5.1)	4.5 (4.2–4.9)
HGB (g/dL)	13.8 (13.4–14.1)	13.1 (12.7–14.2)	13.2 (12.3–13.9)	13.2 (12.8–13.9)	13.4 (12.7–14.3)	13.7 (13.3–14.1)	13.4 (12.6–13.9)	13.7 (12.9–14.2)	13.2 (12.5–13.9)	13.5 (13.1–14.3)	14.2 (13.1–15.5)
PLT (10^3^/μL)	292 (276–301)	264 (242–325)	266 (207–340)	26 (205–287)	298 (227–333)	218***# (175–268)	258 (219–311)	230* (192–285)	253* (218–280)	256 (224–297)	224** (189–278)

Data given as median (25% and 75% percentile), *p < 0.05, **p < 0.01, ***p < 0.001, ****p < 0.0001 indicate significant differences from the control; #*p* < 0.05, ##*p* < 0.01, ###*p* < 0.001, ####*p* < 0.0001 indicate significant differences from the OB 0 patients; ^*p* < 0.05, ^^*p* < 0.01, ^^^*p* < 0.001, ^^^^*p* < 0.0001 indicate significant differences from the OB and OB + MS 0 patients, respectively; ∼*p* < 0.05, ∼∼*p* < 0.01, ∼∼∼*p* < 0.001, ∼∼∼∼*p* < 0.0001 indicate significant differences between OB and OB + MS group according to time before and after laparoscopic sleeve gastrectomy, respectively; BMI, body mass index; WHR, waist-hip ratio; SBP, systolic blood pressure; DBP, diastolic blood pressure; HOMA-IR, homeostatic model assessment of insulin resistance; ALT, alanine transaminase; AST, aspartate transaminase; LDL, low-density lipoprotein; HDL, high-density lipoprotein; TG, triacylglycerol; CRP, C-reactive protein; WBC, white blood cell count; RBC, red blood cell count; HGB, hemoglobin; PLT, platelet count, OB 0 and OB + MS 0 before bariatric surgery, as well as OB 1 and OB + MS 1, 1 month; OB 3 and OB + MS 3, 3 months; OB 6 and OB + MS 6, 6 months; and OB 12 and OB + MS 12, 12 months after bariatric treatment.

The study was approved by the Bioethics Committee of the Medical University of Bialystok (permission numbers: R-I-002/69/2012 and RI-002/475/2019). All research procedures were carried out in accordance with the Declaration of Helsinki and the guidelines for Good Clinical Practice. The informed consents were obtained from all participants of the study.

### Blood collection

The samples from lean and obese patients were taken to EDTA tubes (S-Monovette SARSTEDT) after overnight fasting state. Twenty-four hours before taking blood samples, the patients had not made any intense physical activity. The blood samples were centrifuged at 4,000 rpm in 4 °C for 10 min. A total of 10 μL 0.5 M butylated hydroxytoluene (BHT) for 1 mL plasma sample was added to protect them against oxidation (32). The plasma samples were kept at −80 °C until final tests.

### Laboratory measurements

Plasma glucose, insulin, alanine transaminase (ALT), aspartate transaminase (AST), total cholesterol, low-density lipoprotein (LDL), high-density lipoprotein (HDL) cholesterol, triglycerides (TG), C-reactive protein (CRP), fibrinogen and the full blood count were quantified by using an Abbott analyzer (Abbott Diagnostics, Wiesbaden, Germany). Homeostatic model assessment [HOMA-IR = fasting glucose (mg/dl) × fasting insulin (mU/l)/405] index was estimated according to the formula (33).

### Metalloproteinases assays

The activities of MMPs were measured fluorometrically using MCA-Pro-Leu-Gly-Leu-Dpa(Dnp)-Ala-Arg-NH 2 (MCA is the (7-methoxycoumarin-4-yl) acetate and Dnp is the quencher dinitrophenyl) as the substrate. Incubation time differed for individual MMPs: 40 minutes for metalloproteinase-13 (MMP-13), 1 h for metalloproteinase-2 (MMP-2) and metalloproteinase-7 (MMP-7), 2 h for metalloproteinase-9 (MMP-9), 3 h for metalloproteinase-1 (MMP-1), and 24 h for metalloproteinase-3 (MMP-3), whereas metalloproteinase-11 (MMP-11) was in active form. The fluorescence was measured at 325/393 nm. All assays were performed according to the protocol (34, 35)

### Statistical analysis

GraphPad Prism 9.5.0 for MacOS (GraphPad Software, Inc. La Jolla, United States) was used for statistical analysis. The normality of the distribution was assessed using the Shapiro-Wilk test, while the homogeneity of the variance was checked with Levene’s test. The Mann-Whitney U test was used to compare the study group and the control group. It was also used to compare the study group with obesity and metabolic syndrome versus obese patients without metabolic syndrome within a given time period. For pre- and postoperative comparisons, Kruskal-Wallis ANOVA for pairwise comparisons and Dunn’s *post hoc* test were used. The statistical significance level was set at *p* < 0.05. Multiplicity-adjusted *p*-values were also calculated.

The sample size was determined based on our previous experiment (*n* = 20) using the online calculator Clincalc. Considering the plasma MMP-2 activity and the assay power of 0.8, the minimum number of subjects in the study and control groups should be at least 32.

## Results

### Clinical and laboratory parameters

We observed higher values of BMI, WHR, as well as glucose, HOMA-IR, cholesterol and fibrinogen levels in both groups of individuals with morbid obesity before bariatric treatment as compared with lean ones. HDL levels were diminished. Additionally, individuals in OB + MS O group had increased levels of LDL, TG, CRP, and WBC, as well as hypertension. Glucose homeostasis and lipid profile normalized 12 months after bariatric surgery in both obese study groups. Nevertheless, individuals were overweight in OB group and still obese in OB + MS group 12 months after sleeve gastrectomy (Table 1).

### Collagenases

#### Matrix metalloproteinase 1 (MMP-1)

**FIGURE 1:**
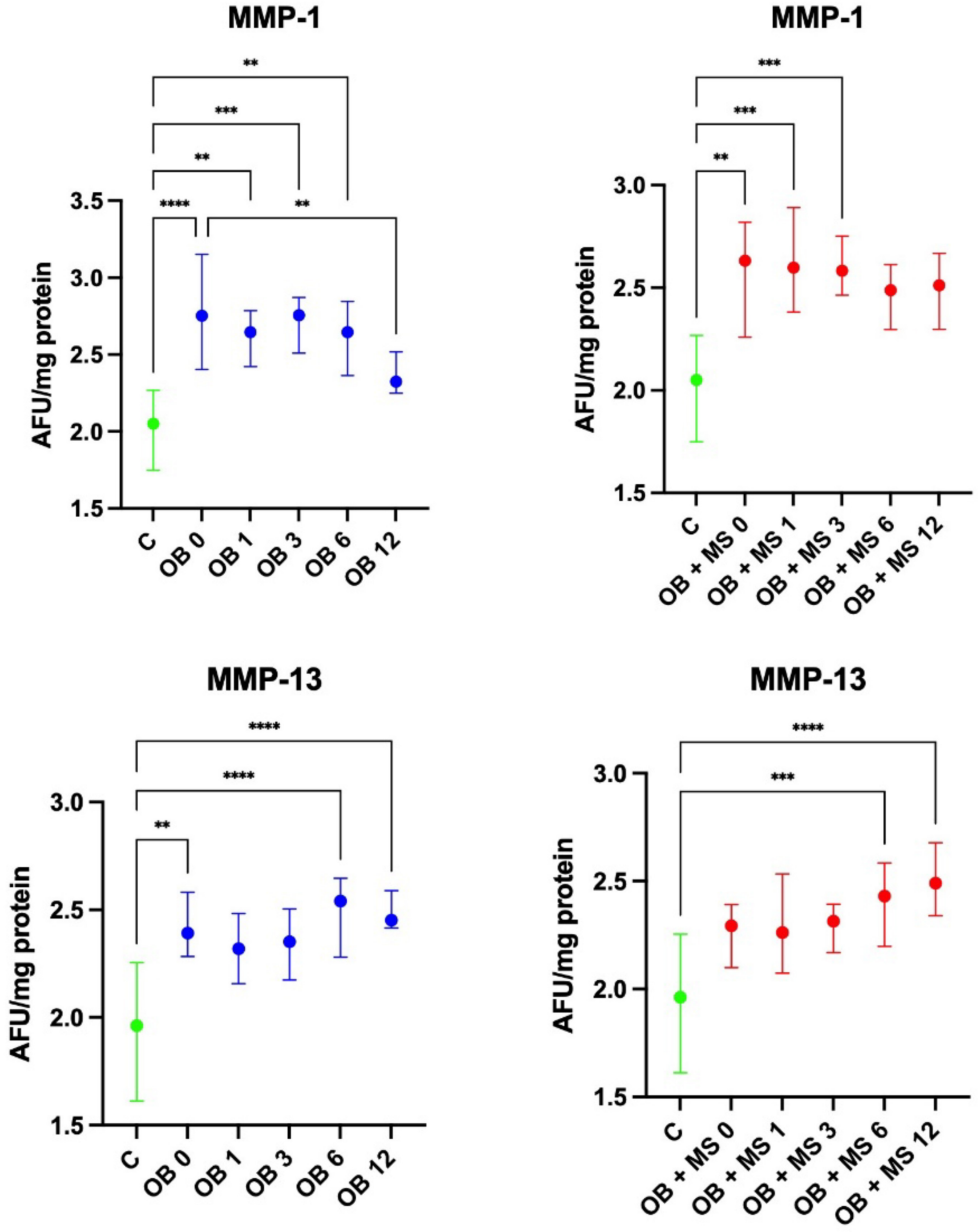
Plasma activity of collagenases: MMP-1 and MMP-13 of the controls, morbidly obese women without metabolic syndrome (OB) and morbidly obese women with metabolic syndrome (OB + MS). Results are presented as median with 25% and 75% percentile; **p < 0.01, ***p < 0.001, ****p < 0.0001; matrix metalloproteinase 1 (MMP-1), matrix metalloproteinase 13 (MMP-13), morbidly obese women without metabolic syndrome and morbidly obese women with metabolic syndrome before (OB 0; OB + MS 0), as well as 1 month (OB 1; OB + MS 1), 3 months (OB 3; OB + MS 3), 6 months (OB 6; OB + MS 6), and 12 months (OB 12; OB + MS 12) after laparoscopic sleeve gastrectomy.

We observed higher plasma activity of MMP-1 in morbidly obese individuals without metabolic syndrome before operation (OB 0: +34%, *p* < 0.0001) in comparison with the controls. Moreover, 1, 3, and 6 months after the bariatric surgery, the activity of MMP-1 was still significantly elevated: OB 1 (+29%, *p* = 0.0028), OB 3 (+34%, *p* = 0.0002) and OB 6 (+29%, *p* = 0.0038). We noticed only 12 months after bariatric surgery that activity of MMP-1 decreased in OB 12 (−16%, *p* = 0.0056) as compared to the OB 0 group (Figure 1).

In morbidly obese individuals with metabolic syndrome the plasma activity of MMP-1 was significantly higher prior to OB + MS 0 (+28%, *p* = 0.0023), as well as 1 month OB + MS 1 (+28%, *p* = 0.0005) and 3 months OB + MS 3 (+27%, *p* = 0.0005) after the bariatric surgery as compared to the controls (Figure 1).

#### Matrix metalloproteinase 13 (MMP-13)

In plasma activity of MMP-13 was higher in morbidly obese individuals without metabolic syndrome before (OB 0: +22%, *p* = 0.0035), as well as six (OB 6: +29%, *p* < 0.0001) and 12 months (OB 12: +25%, *p* < 0.0001) after bariatric treatment in comparison with lean patients (Figure 1).

In morbidly obese individuals with metabolic syndrome, we observed increased the plasma activity of MMP-13 only after bariatric surgery: in OB + MS 6 (+24% *p* = 0.0005) and twelve OB + MS 12 (+27%, *p* < 0.0001) groups (Figure 1).

### Gelatinases

#### Matrix metalloproteinase 2 (MMP-2)

**FIGURE 2:**
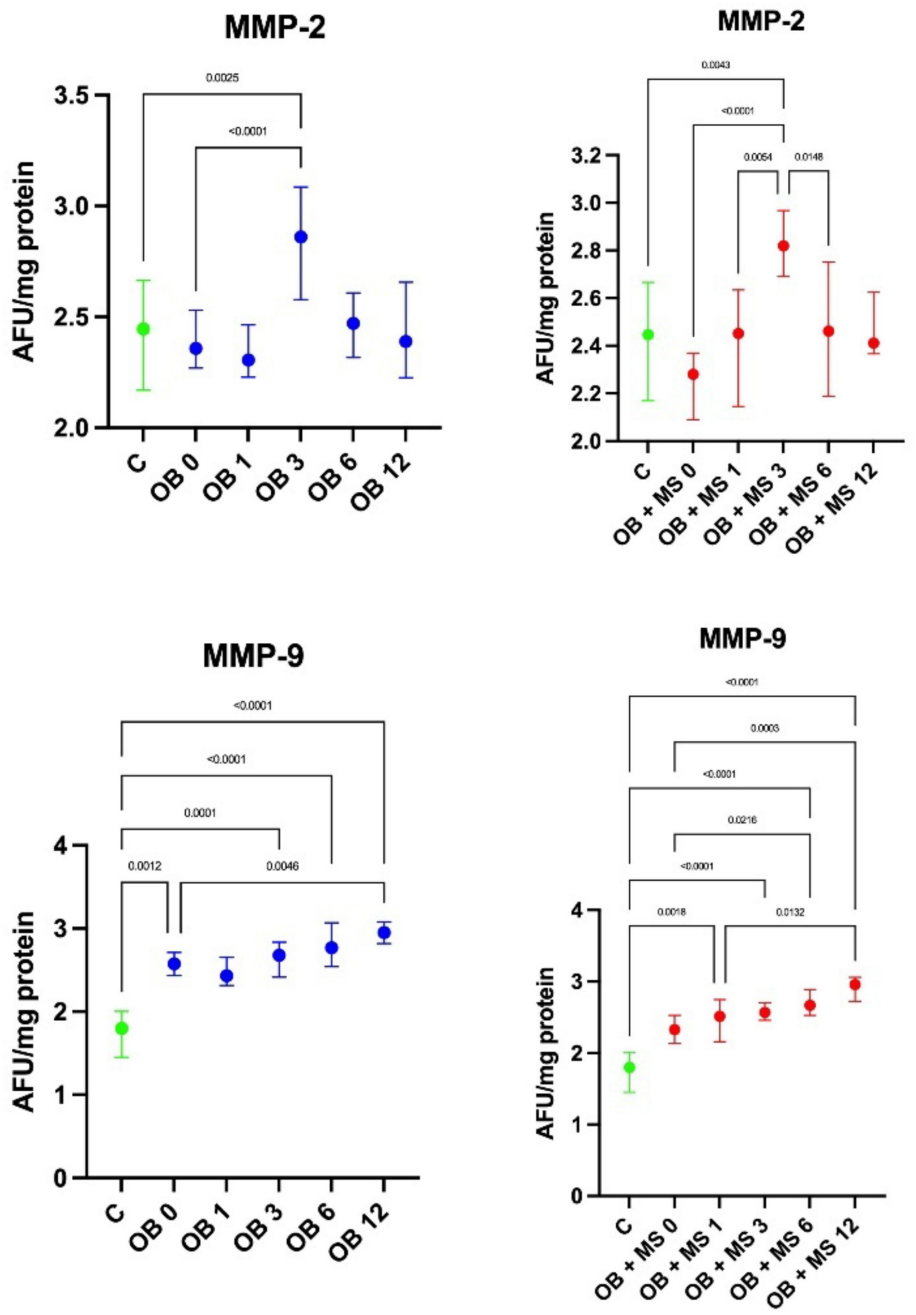
Plasma activity of gelatinases: MMP-2 and MMP-9 of the controls, morbidly obese women without metabolic syndrome (OB) and morbidly obese women with metabolic syndrome (OB + MS). Results are presented as median with 25% and 75% percentile; *p < 0.05, **p < 0.01, ***p < 0.001, ****p < 0.0001; matrix metalloproteinase 2 (MMP-2), matrix metalloproteinase 9 (MMP-9), morbidly obese women without metabolic syndrome and morbidly obese women with metabolic syndrome before (OB 0; OB + MS 0), as well as 1 month (OB 1; OB + MS 1), 3 months (OB 3; OB + MS 3), 6 months (OB 6; OB + MS 6), and 12 months (OB 12; OB + MS 12) after laparoscopic sleeve gastrectomy.

There were no statistically significant differences in activity of plasma MMP-2 in morbidly obese women without metabolic syndrome prior to bariatric surgery as compared to the controls. We found only greater MMP-2 activity three months after sleeve gastrectomy (OB 3: +17%, *p* = 0.0025) than in the controls. The activity of MMP-2 was also higher in OB 3 (+21%, *p* < 0.0001) than the OB 0 group (Figure 2).

We observed similar results in women with obesity and metabolic syndrome. Plasma activity of MMP-2 was increased in OB + MS 3 (+15%, *p* = 0.0043; +24, *p* < 0.0001) in comparison with the lean controls and OB + MS 0 group, respectively (Figure 2).

#### Matrix metalloproteinase 9 (MMP-9)

We noticed markedly higher plasma activity of MMP-9 in morbidly obese individuals without metabolic syndrome before operation (OB 0: +43%, *p* = 0.0012) in comparison with the controls. Moreover, 3, 6, and 12 months after the bariatric surgery, the activity of MMP-9 was still significantly elevated: OB 3 (+49%, *p* = 0.0001), OB 6 (+54%, *p* < 0.0001) and OB 12 (+64%, *p* < 0.0001). Interestingly, MMP-9 activity was greater in OB 12 (+15%, *p* = 0.0046) than OB 0 group (Figure 2).

In morbidly obese individuals with metabolic syndrome plasma activity of MMP-9 did not differ between OB + MS 0 and the controls. MMP-9 activity was significantly higher 1 month (OB + MS 1: +40%, *p* = 0.0018), 3 month (OB + MS 3: +43%, *p* < 0.0001), 6 month (OB + MS 6: +48%, *p* < 0.0001), and 13 months (OB + MS 12: +64%, p < 0.0001) after bariatric surgery than in the controls. Furthermore, we observed an increase in MMP-9 activity in OB + MS 6 (+15%, *p* = 0.0216) and OB + MS 12 (+27%, *p* = 0.0003) compared with OB + MS 0 group (Figure 2).

### Stromelysins

#### Matrix metalloproteinase 3 (MMP-3)

**FIGURE 3:**
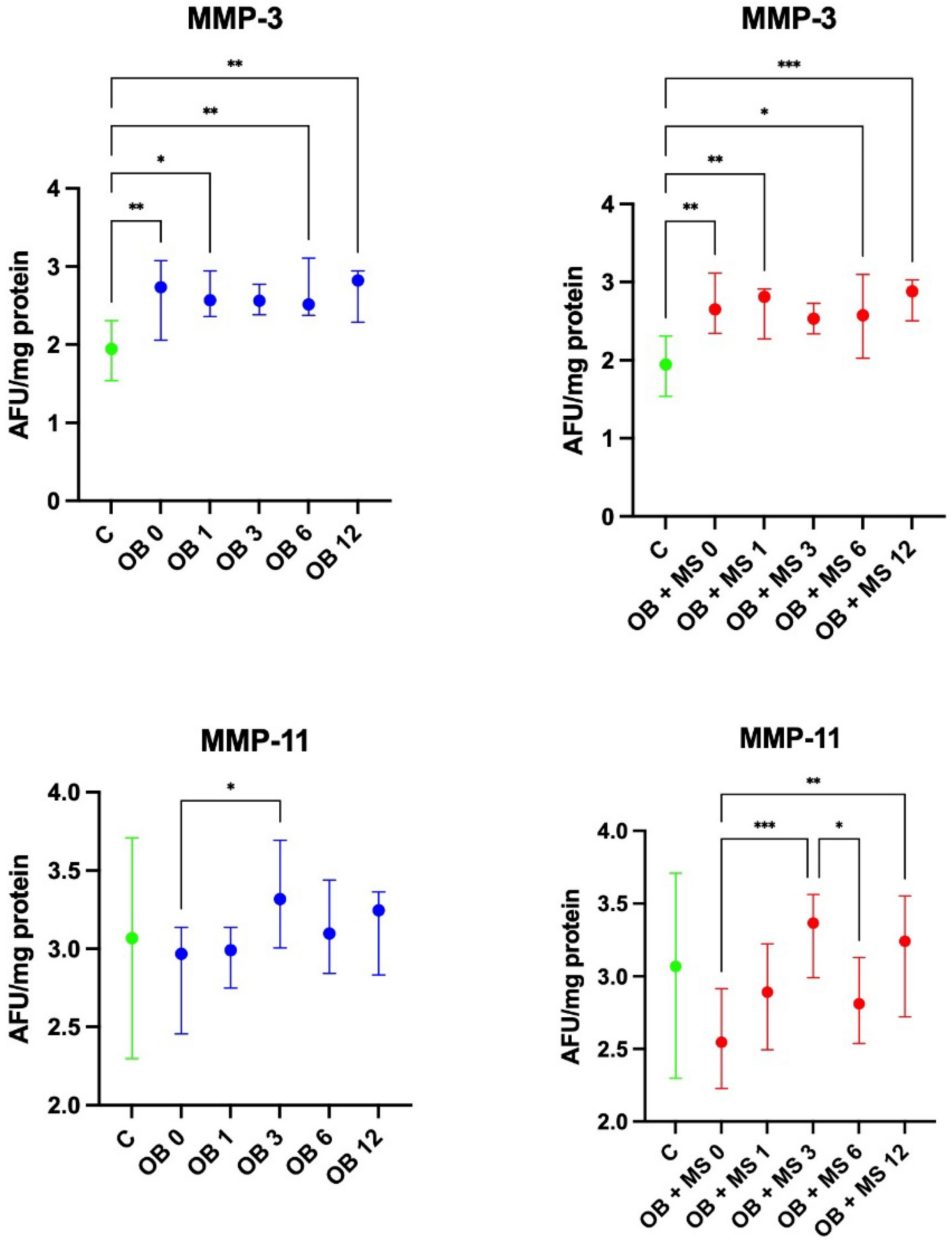
Plasma activity of stromelysins: MMP-3 and MMP-11 of the controls, morbidly obese women without metabolic syndrome (OB) and morbidly obese women with metabolic syndrome (OB + MS). Results are presented as median with 25% and 75% percentile; *p < 0.05, **p < 0.01, ***p < 0.001; matrix metalloproteinase 3 (MMP-3),, matrix metalloproteinase 11 (MMP-11), morbidly obese women without metabolic syndrome and morbidly obese women with metabolic syndrome before (OB 0; OB + MS 0), as well as 1 month (OB 1; OB + MS 1), 3 months (OB 3; OB + MS 3), 6 months (OB 6; OB + MS 6), and 12 months (OB 12; OB + MS 12) after laparoscopic sleeve gastrectomy.

Plasma activity of MMP-3 in morbidly obese individuals without metabolic syndrome was statistically higher before operation (OB 0: +41%, *p* = 0.0056), as well as 1 month (OB 1: + 32%, *p* = 0.0128), 6 month (OB 6: + 29%, *p* = 0.0056), and 12 months (OB 12: + 45%, *p* = 0.0022) after bariatric surgery in comparison with the controls (Figure 3).

Similarly, in morbidly obese individuals with metabolic syndrome, MMP-3 activity was greater before operation (OB + MS 0: +36%, *p* = 0.0032), as well as 1 month (OB + MS 1: +45%, *p* = 0.0069), 6 month (OB + MS 6: +32%, *p* = 0.0433), and 12 months (OB + MS 12: +48%, *p* = 0.0005) after bariatric surgery than in the controls (Figure 3).

#### Matrix metalloproteinase 11 (MMP-11)

There were no statistically significant differences in activity of MMP-11 in plasma of morbidly obese women without metabolic syndrome compared with the controls. However, MMP-11 activity was higher in OB 3 (+12%, *p* = 0.0146) that the OB 0 group (Figure 3).

The plasma activity of MMP-11 did not differ between morbidly obese individuals with metabolic syndrome and the lean controls. Nevertheless, its activity increased 3 month (OB + MS3: +32%, *p* = 0.0001) and 12 months (OB + MS 12: +27%, *p* = 0.009) after bariatric treatment as compared to OB + MS 0 (Figure 3).

### Matrilysin

#### Matrix metalloproteinase 7 (MMP-7)

**FIGURE 4:**
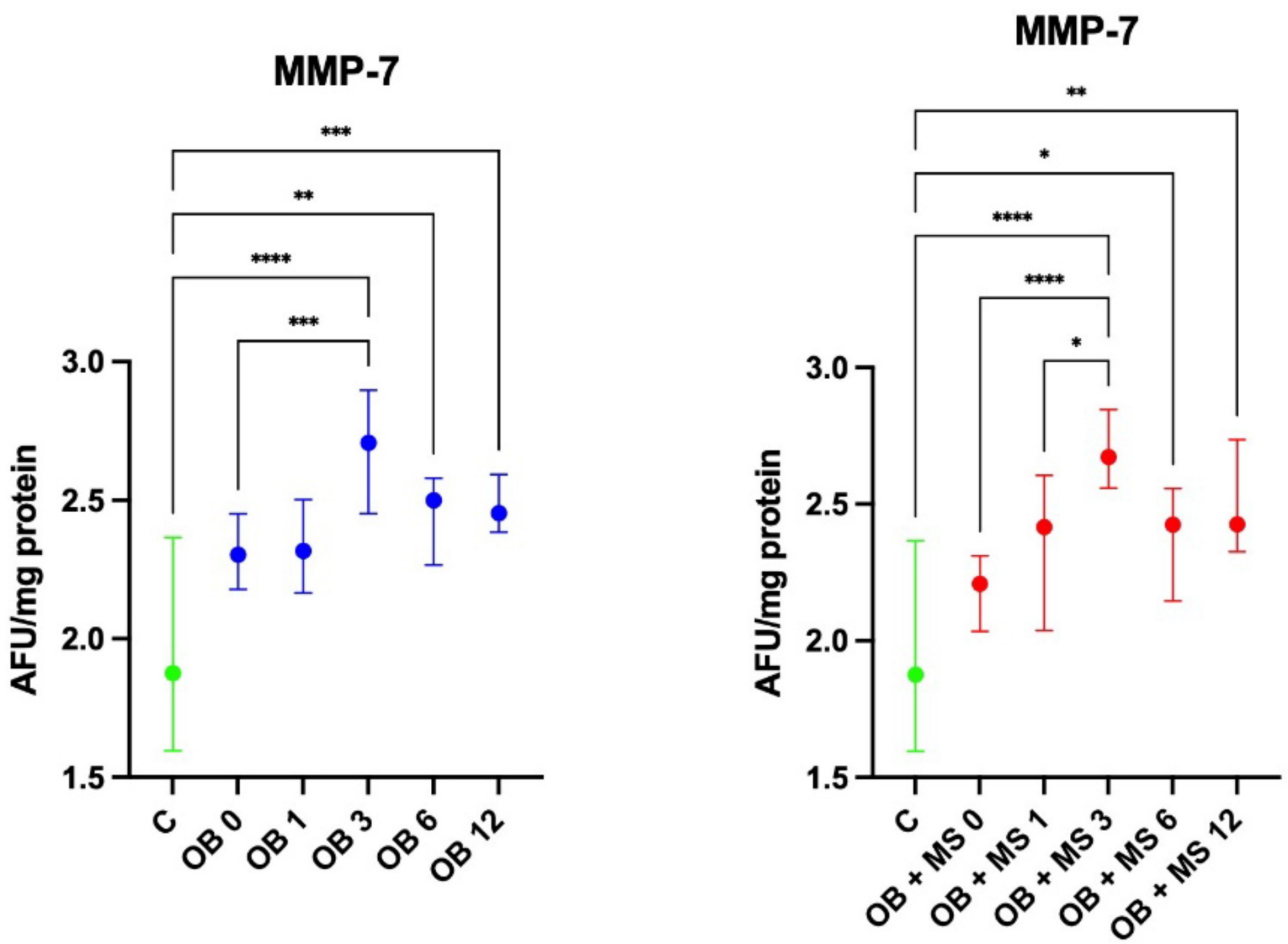
Plasma activity of matrilysin: MMP-7 of the controls, morbidly obese women without metabolic syndrome (OB) and morbidly obese women with metabolic syndrome (OB + MS). Results are presented as median with 25% and 75% percentile; * *p* < 0.05, **p < 0.01, ***p < 0.001, ****p < 0.0001; matrix metalloproteinase 7 (MMP-7), morbidly obese women without metabolic syndrome and morbidly obese women with metabolic syndrome before (OB 0; OB + MS 0), as well as 1 month (OB 1; OB + MS 1), 3 months (OB 3; OB + MS 3), 6 months (OB 6; OB + MS 6), and 12 months (OB 12; OB + MS 12) after laparoscopic sleeve gastrectomy.

We did not find differences in activity of MMP-7 in plasma of morbidly obese women without metabolic syndrome before bariatric operation compared with lean controls. However, activity of MMP-7 was greater in OB 3 (+44%, *p* < 0.0001), OB 6 (+33%, *p* = 0.0018), and OB 12 (+31%, *p* = 0.0005) groups than in the controls. Moreover, MMP-7 activity was higher in OB 3 (+18%, *p* = 0.0002) in comparison with OB 0 (Figure 4).

We obtained similar results in morbidly obese individuals with metabolic syndrome: plasma activity of MMP-7 was increased in OB + MS 3 (+43%, *p* < 0.0001), OB + MS 6 (+29%, *p* = 0.0109) and OB + MS 12 (+29%, *p* = 0.0014) groups as compared to the lean controls. Activity of MMP-7 in plasma of OB + MS 3 (+21%, *p* < 0.0001) was also higher than OB + MS 0 group (Figure 4).

Additionally, we compared above mentioned parameters between morbidly obese individuals without metabolic syndrome (OB) and morbidly obese individuals with metabolic syndrome (OB + MS) before, as well as 1, 3, 6 and 12 months after bariatric treatment. We observed lower plasma activities of MMP-9 (−10%, *p* = 0.008), MMP-11 (−14%, *p* = 0.0248) and MMP-13 (−4%, *p* = 0.0455) in morbidly obese individuals with metabolic syndrome in comparison with morbidly obese individuals without metabolic syndrome before bariatric surgery. Moreover, the plasma activity of MMP-11 smaller in OB + MS 6 (−9%, *p* = 0.0265) than OB 6, whereas plasma activity of MMP-1 was greater in OB + MS 12 (+8%, *p* = 0.0419) as compared to OB 12.

### Correlations

In morbidly obese individuals without metabolic syndrome MMP-1 correlated positively with LDL (*R* = 0.433, *p* = 0.019) before surgery (OB 0) and 6 month after treatment (OB 6) (*R* = 0.505, *p* = 0.01).

In morbidly obese individuals with metabolic syndrome MMP-2 correlated negatively with glucose (*R* = −0.452, *p* = 0.023) in OB + MS 3 and (*R* = −0.535, *p* = 0.012) in OB + MS 6 study group.

MMP-3 correlated positively with HDL (*R* = 0.598, *p* = 0.003) in OB 1 group. We also found positive correlations MMP-3 with HOMA-IR (*R* = 0.537, *p* = 0.01) in OB + MS 1 and LDL (*R* = 0.487, *p* = 0.029) OB + MS 6.

In morbidly obese individuals without metabolic syndrome MMP-11 correlated with LDL: positively (*R* = 0.39, *p* = 0.036) before surgery (OB 0) and negatively (*R* = −0.463, *p* = 0.034) twelve months after treatment (OB 12).

MMP-13 correlated positively with WHR (*R* = 0.512, *p* = 0.038) in OB 12 group.

## Discussion

As obesity is one of the main risk factors of metabolic and cardiovascular diseases, it seems crucial to understand its pathophysiology. In this study, we demonstrated many disturbances in the MMPs homeostasis in women with morbid obesity. The plasma activities of collagenases (MMP-1 and MMP-13), gelatinases (MMP-9), and stromelysins (MMP-3) were significantly increased in morbidly obese women without metabolic syndrome before laparoscopic sleeve gastrectomy, whereas in women with morbid obesity and metabolic syndrome we observed higher activities of MMP-1 and MMP-3 than in the lean healthy controls. After bariatric treatment, only the activity of some plasma MMPs partially normalized in both obese groups studied.

The main role of MMPs is the degradation of ECM proteins, which facilitates cell migration and releases growth factors in both normal and pathological states. Under physiological conditions, MMPs are involved in the regulation of embryogenesis, angiogenesis, immunological, metabolic (36), wound healing processes, and also participate in the formation of cell receptors (37). However, excessive activity of MMPs is observed in atherosclerosis, cardiovascular diseases, vascular complications of diabetes and cancer (13, 37, 38).

Expression, secretion and activity of MMPs are tightly controlled (36). In general, they are produced at low levels but during active tissue remodeling their secretion is rapidly induced. Most MMPs are secreted as inactive proenzymes and activated by other proteinases, plasmin or oxygen free radicals. Indeed, in our previous study, we found increased oxidative stress and impaired antioxidant barrier in morbidly obese patients (39, 40). In turn, MMPs are inhibited through interactions with tissue inhibitors of MMPs (TIMPs) (11). Although we did not assess TIMPs levels in this study, it is noteworthy that they are associated with adipose tissue development. Moreover, we observed changes in plasma MMPs activity in individuals with obesity, it should be emphasized that the level of MMPs in plasma is not specific to adipose tissue.

Matrix metalloproteinases are involved in the development of obesity through the ability to modulate the process of adipogenesis. Modifications of adipose tissue occurring in the obesity progression are associated with migration, remodeling and proteolysis of ECM, as well as hypoxia and fibrosis (11, 16). In the development of obesity, during rapid adipose tissue expansion progressive hypoxia contributes to its dysfunction (41–43). The consequence of hypoxia is fibrosis, which in turn, by decreasing flexibility of ECM (44, 45), is a major factor leading to metabolic abnormalities in adipose tissue (44–46). During the process of adipose tissue fibrosis, excessive accumulation of collagen 6 (COL6) occurs in the ECM. As a result, a rigid and inflexible matrix is formed, which is a physical barrier against further enlargement of adipose tissue, and the developing mechanical stress leads to local and systemic metabolic disorders (47, 48). It has been observed that COL6 deficient mice display destabilized ECM and diminished fibrosis process in adipose tissue, as well as improved insulin sensitivity (49). Indeed, systemic insulin resistance develops as a result of local inflammation which is enhanced by impaired collagen accumulation in remodeling process of the ECM (15, 50). Overexpressed MMP-14 in adipose tissue of obese mice digests COL6 and, indirectly, by production of endotrophin, stimulates fibrosis, as well as metabolic disorders. Endotrophin, a newly identified adipokine, leads to impaired lipid and glucose metabolism, decreased energy expenditure and systemic insulin resistance (51, 52). Nevertheless, Xin at al. (16) discovered that overexpression of MMP14 in early-stage of obesity prevents abnormal accumulation of fibrotic proteins and promotes healthy expansion of adipose tissue. In our study, we observed higher plasma MMPs activity in women with morbid obesity compared to the controls, which decreased 12 months after bariatric treatment. The persistently elevated activity of MMPs after bariatric surgery in women with morbid obesity may have been due to the long duration of obesity. Furthermore, secretion of proinflammatory factors and production of elevated levels of free fatty acids by adipose tissue intensify lipotoxicity in other organs, like muscles, heart or liver (53, 54). In mice fed a high fat diet inactivation of the MMP-3 gene led to extended development of adipose tissue. Moreover, MMP-3 deficient mice had decreased plasma triglyceride levels in comparison with the wild-type controls mice. Probably, MMP-3 deficiency contributes to modification of circulating plasma triglycerides to intracellular lipid droplets, their storage form in adipose tissue. It is associated with enhanced lipoprotein lipase activity and/or synthesis (55). In our study we found positive correlations MMP-1 and MMP-11 with LDL in morbidly obese individuals. Higher body weight was observed after feeding high-fat diet also in MMP-11 deficient mice. Adipocytes in MMP -11 deficient mice adipose tissue showed hypertrophy, however, it is not clear whether this is directly related to MMP-11 (56). Also, increased activity of MMP-2 and MMP-9 could be caused by disturbances of lipid metabolism in obese people. Derosa et al. (57) showed higher plasma concentrations of MMP-2 and MMP-9 in patients with dyslipidemia. Oxidatively modified LDL particles derived from atherosclerotic plaque macrophages stimulate the expression of MMP-9 and downregulate the expression of the TIMP-1 inhibitor. However, this effect is neutralized by HDL (58). Nowadays, there are some contradictions in studies regarding the levels of MMP-2 and MMP-9 in obesity (59, 60). MMP-2 enhances adipocyte differentiation (61). Elevated expression of MMP-2 was described in adipose tissue of obese mice (12, 62). Derosa et al. (57) observed higher concentrations of MMP-2 and MMP-9 in obese individuals compared to lean people. Also in the group of obese children and adolescents, the concentration of MMP-9 was increased, but the concentration of MMP-2 was decreased (63). Moreover, the relationship between gelatinases and the pathogenesis of hypertension and T2DM is unclear. Increased activities and concentrations of MMP-2 and MMP-9 have been demonstrated in individuals with primary and isolated systolic hypertension and cardiovascular compications (57, 64, 65). In the early phase of the development of hypertension, an increase in the activity of MMPs may be beneficial, as it increases the vasodilation and, as a result, reduces systolic pressure. Nevertheless, the long-term increase in MMPs activity contributes to the permanent remodeling and stiffening of blood vessels (66). Although in our study the majority of individuals from the OB + MS group suffered from hypertension and T2DM, we only observed increased activity of MMP-9 in the OB group before bariatric surgery. This could be the result of taking antihypertensive and anti-diabetic drugs in the OB + MS group and their influence on the activity of MMPs cannot be excluded. Moreover, MMP-2 correlated negatively with glucose in OB + MS 3 and in OB + MS 6 study group. The obtained results of gelatinases assays may have been also affected by disturbances in carbohydrate metabolism. In the course of diabetes, there is an imbalance between the concentration of MMPs and their inhibitors (67). Elevated levels of MMP-2 and MMP-9 in serum of patients with hypertension and T2DM may reflect early remodeling changes in the vascular ECM (38). In study conducted on rats, with streptozotocin and a high-fat diet induced diabetes, it was observed that overexpression of the cardiac IGF-1 receptor (IGF-1R) led to an increase in the expression of MMP-2 and MMP-9, as well as type I collagen in cardiac fibroblasts (20). Interestingly, in our study carbohydrate metabolism and blood pressure improved partially, while the activities of MMP-2 and MMP-9 were elevated in both study groups after bariatric treatment. Similar results was obtained after gastric bypass surgery (25). Wu et al. (68) suggested that bariatric surgery improve glucose homeostasis in obese patients with T2DM regardless the MMP-2 and MMP-9 pathways, because they found no difference in gelatinase concentrations in obese individuals before and after bariatric surgery. In our study, it is worth noting that the OB individuals were overweight and participants in OB + MS group were still obese 12 months after bariatric surgery. We suppose that 12 months after bariatric treatment is insufficient to regulate MMPs balance. It is not surprise, because increased oxidative stress is one of main process leading to disturbances in MMPs homeostasis. In our previous study we observed abnormalities in redox homeostasis in individuals with morbid obesity similar to MMPs results, continuing many months after surgery. Bariatric treatment led to the resolution of metabolic complications of obesity, but disturbances of the antioxidant barrier persisted in obese women with metabolic syndrome (39).

## Limitations

Our study has several limitations. First, we assessed only the activity of plasma MMPs. However, extracellular matrix (ECM) remodeling cannot be fully evaluated without analyzing the activity of tissue inhibitors of metaloproteinases (TIMPs). This constitutes the major limitation of the study. Second, as we measured only circulating MMP levels, we cannot draw conclusions regarding their direct involvement in obesity-related organ complications. Third, our analyses were not adjusted for potential confounding factors such as medication use, inflammatory status, or variability in weight loss. In addition, although we observed changes in plasma MMPs activity in individuals with obesity, it should be emphasized that the level of MMPs in plasma is not specific to adipose tissue and may depend on many other factors. Further studies on the effects of bariatric surgery on mRNA levels, protein expression and specific activity of MMPs/TIMPs are needed in the target tissues of individuals with obesity. This study being done on a female only cohort. Since the production/secretion of MMPs can be gender-dependent, studies in the male population are also necessary.

## Conclusion

Obesity is associated with increased activities of plasma matrix metalloproteinases. Although bariatric surgery improves MMPs homeostasis of individuals with obesity, it remained disturbed even 12 months after bariatric treatment. In general, the activity of MMPs did not differ between obese individuals without and with the metabolic syndrome. However, in individuals with metabolic syndrome, disturbances in the activity of MMPs persisted longer after bariatric surgery. Further long-term studies are needed to validate our findings.

## Data Availability

The raw data supporting the conclusions of this article will be made available by the authors, without undue reservation.
